# Use of tocilizumab in a patient with severe COVID-19 in a Teaching Hospital in Ghana: a case report

**DOI:** 10.11604/pamj.supp.2020.37.1.25851

**Published:** 2020-10-29

**Authors:** Yasmine Oladele Hardy, Kojo Awotwi Hutton-Mensah, Divine Aseye Yao Amenuke, James Amoah-Dankwah

**Affiliations:** 1 Department of Medicine, Komfo Anokye Teaching Hospital, Box 1934, Kumasi, Ghana

**Keywords:** Tocilizumab, COVID-19, interleukin-6, sub-Saharan Africa, case report

## Abstract

The outbreak of coronavirus disease 2019 (COVID-19) in December 2019 has rapidly spread globally with significant negative impact on health. There is an urgent need for a drug or vaccine certified for treating and preventing COVID-19 respectively. Tocilizumab, an interleukin-6 monoclonal receptor antibody, has been used in some centers for mitigating the severe inflammatory response seen in patients with severe COVID-19 with encouraging results. To the best of our knowledge, reports detailing the outcomes of patients with severe COVID-19 undergoing treatment with tocilizumab are sparse in sub-Saharan Africa. We describe the clinical and laboratory profile, chest Computed Tomography (CT) scan findings and clinical outcome in a Ghanaian patient with severe COVID-19 pneumonia treated with tocilizumab. A 54-year-old hypertensive male presented with fever, productive cough, pleuritic chest pain and breathlessness. He tested positive for severe acute respiratory syndrome coronavirus 2 (SARS-CoV-2) by polymerase chain reaction analysis done on a nasopharyngeal swab sample. His respiratory symptoms worsened while on admission despite receiving standard of care. His C-reactive protein (CRP) was elevated to 80.59mg/L and chest CT scan findings were indicative of severe COVID-19 pneumonia. He was treated with a single 400mg dose of intravenous tocilizumab with a positive clinical outcome, rapid decline in CRP and improvement in chest CT findings. Our experience shows that tocilizumab shows great promise as drug therapy for COVID-19 pneumonia.

## Introduction

In December 2019, a cluster of pneumonia cases, caused by a newly identified β-coronavirus (SARS-CoV-2), occurred in Wuhan, China [1]. This outbreak has rapidly scaled up to become a pandemic that has stretched the resources of health systems to breaking point [1]. The presentation is mainly mild or asymptomatic but more severe cases of hypoxia, sepsis, acute respiratory distress syndrome, respiratory and other organ failure may occur [1,2]. The cytokine release syndrome (CRS), which has been reported in severe COVID 19 cases, is caused by a dysregulated host immune response mediated by pro-inflammatory cytokines with cellular infiltration, widespread lung inflammation and damage; and multi-organ failure [2]. Interleukin-6 (IL-6) stimulates production of B and cytotoxic T cells and is thus a key player in the CRS [3]. Currently there is no approved drug for treating COVID-19. It can however be deduced that therapies aimed at reducing the levels of pro-inflammatory cytokines might be of benefit to patients with severe COVID-19. Various clinical trials on a number of drugs are ongoing, including tocilizumab, a monoclonal antibody against the IL-6 receptor, which is also used in treatment of chronic conditions like rheumatoid arthritis as well as the CRS seen in patients receiving chimeric antigen T-cell therapy [1,4]. A number of studies from China, United States of America and parts of Europe [5-7] have reported positive outcomes with the use of tocilizumab in managing critically ill patients, including mechanically ventilated patients. To the best of our knowledge reports from sub-Saharan Africa are scanty. Studies are needed from within sub-Saharan Africa as the drug response may vary from that seen in populations outside the region due to differences in genetic makeup. We report our experience with tocilizumab in a patient with severe COVID-19 pneumonia at the Highly Infectious Isolation Unit (HIIU) of the Komfo Anokye Teaching Hospital (KATH), Kumasi in the Ashanti Region of Ghana.

## Patient and Observation

**Figure 1:**
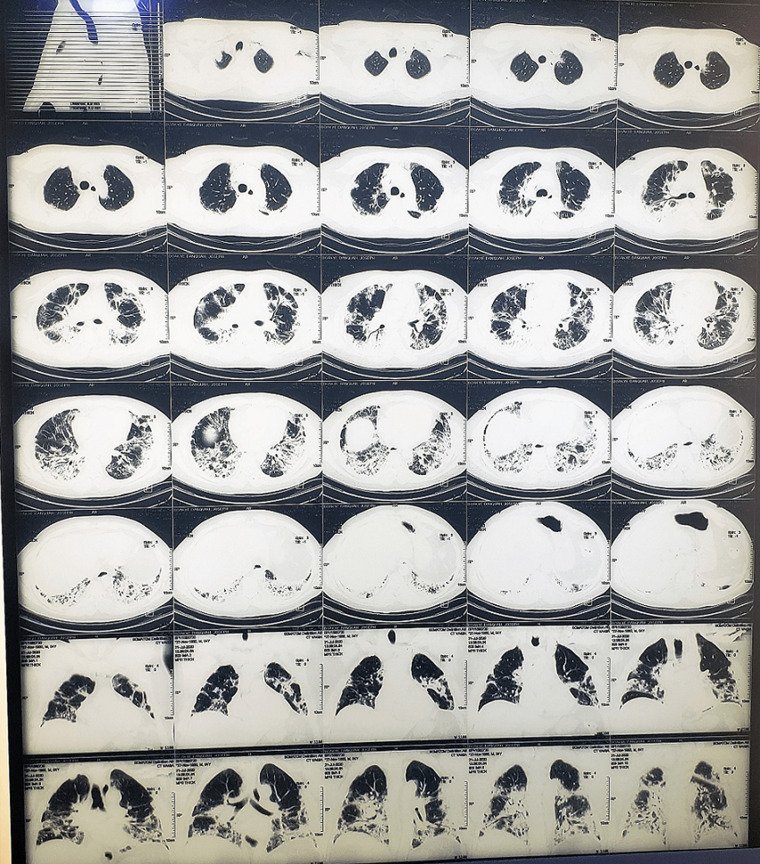
computed tomography pulmonary angiogram on day 7

**Figure 2:**
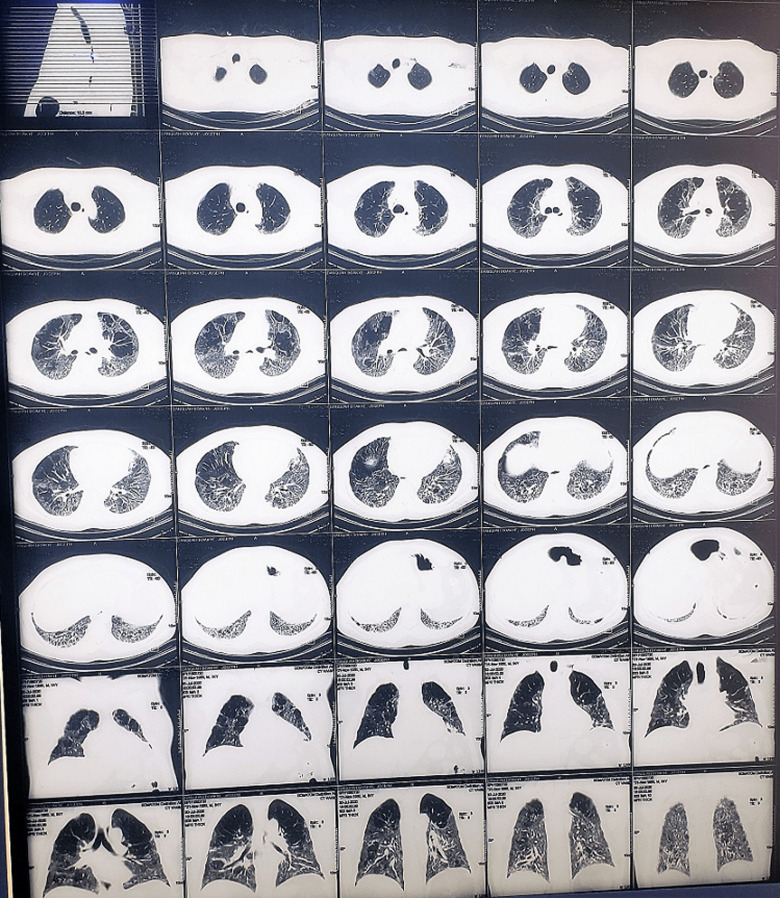
computed tomography pulmonary angiogram on day 16

A 54-year-old male who is a known hypertensive compliant on medications, was admitted at the HIIU as a case of COVID-19 pneumonia (positive SARS-CoV-2 polymerase chain reaction analysis on a nasopharyngeal swab sample). He had a 3-week history of fever, cough productive of yellowish, non-bloody sputum and pleuritic chest pain. He also had a week´s history of progressively worsening breathlessness. There was associated sore throat, headache and myalgia. He had no contact with a confirmed COVID-19 patient. Initial chest computer tomography (CT) scan at the onset of symptoms showed features suggestive of mild COVID-19 pneumonia. At presentation his symptoms had worsened. His temperature was 36.4°C, pulse rate was 58 beats/min, respiratory rate 30cycles/min, blood pressure 145/96mmHg and oxygen saturation (SpO_2_) 93% on oxygen delivered via non-renon-rebreather mask at 15Liters/min. His full blood count showed leukocytosis with neutrophilia (Table 1). Sinus bradycardia and a corrected QT interval (QTc) of 0.446s was seen on initial ECG with a repeat QTc on completion of hydroxychloroquine being 0.426s. Cultures for blood and urine were negative. Sputum gene Xpert was negative for Mycobacterium tuberculosis. He was initiated on empiric intravenous ceftriaxone 2g twice daily for 72 hours, intravenous dexamethasone 6mg daily for 10 days and subcutaneous enoxaparin 80mg twice daily. He was also given oral hydroxychloroquine 200mg three times daily for 10 days, oral azithromycin 500mg stat, then 250mg daily x 4; zinc 20mg daily (according to Ghana´s Standard Treatment Guidelines for COVID-19) oral zinc 20mg daily x 21 and oral multivitamins 1 daily x 30. On day 7 of admission, 15 days after the initial chest CT scan was taken, his symptoms had worsened further with marked breathlessness on minimal exertion and easy fatiguability. His respiratory rate was 40 cycles/min. SpO_2_on oxygen given via non-rebreather mask at 15L/min was 94% and 85% in room air. CT pulmonary angiogram (CTPA) was requested due to suspicion of pulmonary embolism. It showed severe worsening of bilateral consolidation and ground glass densities with fibrotic changes in the lung bases on both sides. There was no evidence of pulmonary embolism (Figure 1). On day 11 of admission, his C-reactive protein (CRP) came out as 80.59mg/L (Table 1). A single dose of 400mg of tocilizumab reconstituted with 80mls of 0.9% of normal saline solution was administered via a perfuser over 60 minutes on day 12; on account of deteriorating respiratory symptoms, hypoxia, an elevated CRP (all indicators of CRS) and worsening chest CT scan findings. His respiratory symptoms gradually improved. CRP done on day 15 was 4.57mg/L and a repeat chest CTPA done on day 16 showed marked improvement with significant loosening of the bilateral consolidations and improvement in bilateral ground glass densities (Figure 2). There was however a bilateral base of fibrotic changes for which oral pirfenidone was initiated for 3 weeks. His lymphocyte count also increased from 2.06 x10^3^/microliter to 2.78 x10^3^/microliter. On day 21 of admission he was weaned off oxygen. His SpO_2_in room air was 96%. He was discharged on day 23 for review at the outpatient clinic and is presently doing very well clinically.

**Table 1:** laboratory results of the patient

Lab findings	Day 0	Day 11	Day 15
Hb (11.5- 16.5g/dL)	13.3	12.4	12.9
MCV (80.0-100.0 fL)	84.5	89.0	85.8
MCH (26.0-38.0pg)	33.3	28.4	28.1
WBC (4.00-10.00 x103/µL)	12.79	10.56	9.64
Neutrophils (1.50-7.00 x 103/µL)	10.23	7.69	6.19
Lymphocytes (1.00-3.70 x103/µL)	1.74	2.06	2.78
Platelet count (140-400 x103/µL)	322	428	412
ALT (0.0-41.0 U/L)	122.9	57.6	52.7
AST (0.0-40.0 U/L)	78.9	21.6	40.3
Albumin (35.0-52.0 g/L)	39.1	37.2	44.1
Urea (2.50-8.30 mmol/L)	8.0	4.9	4.2
Creatinine (44-80 micromol/L)	107	84	97
HIV antibodies	Negative		
Hepatitis B surface antigen	Negative		
Hepatitis C antibody	Negative		
Erythrocyte sedimentation rate(mm/hr)	120		
CRP(mg/L)		80.59	4.57

Abbreviations: Hb, hemoglobin; WBC, white cell count; PLT, platelets; ALT, alanine aminotransferase; AST, aspartate aminotransferase

## Discussion

In this report we have presented a patient in sub-Saharan Africa who had clinical and laboratory evidence of pronounced inflammation indicative of CRS. He however responded to intravenous tocilizumab with a positive clinical outcome as well as significant, rapid improvement in CRP and chest CT scan findings. These findings buttress what has been reported by other authors [1,4]. C-reactive protein (CRP) which is produced as a result of the increased synthesis of pro-inflammatory cytokines activates the complement immune response and is a reliable inflammatory marker of disease severity and even mortality [4,8]. Thus both IL-6 and CRP are among the major indicators of the CRS [3,8]. We used CRP as a surrogate inflammatory marker since we did not have the logistics to measure IL-6 levels. Our patient had high initial CRP levels with a remarkable decline following tocilizumab administration. Kewan and his co-authors also had similar findings in their study [1]. Due to the role of IL-6 in activating the innate immune system, it is expected that its blockade would predispose patients to superinfections [9]. Other documented side effects of this drug include headaches, hypertension, liver injury with a concurrent rise in alanine aminotransferase; and upper respiratory tract infections [3]. However, our patient had a favorable clinical outcome void of any infection or adverse event following tocilizumab use in contrast to what was reported in other studies [3,9]. This may be because a lower dose of 400mg was used in our study in contrast to the total higher dose (800mg) used in the aforementioned studies. This implies that 400mg tocilizumab may produce similar outcomes with fewer side effects compared to higher doses [1]. Post-inflammatory pulmonary fibrosis is a feature of severe COVID-19 pneumonia in the early recovery phase and can have a negative effect on quality of life [10]. Pirfenidone, an antifibrotic agent has been used successfully as treatment [10]. Our patient was managed with this drug and was doing well clinically 2 weeks after discharge. A repeat chest CT scan would be done after completion of pirfenidone to assess for resolution of fibrosis.

## Conclusion

The findings from this case report are encouraging and add to the emerging literature on treatment of COVID-19 with tocilizumab. However, large multicenter, randomized controlled trials on tocilizumab, and other IL-6 blocking agents are awaited (especially in Africa) to throw more light on the efficacy and safety of tocilizumab as well as the timing of administration.
